# The RNA degradation pathway is involved in PPARα-modulated anti-oral tumorigenesis

**DOI:** 10.1051/bmdcn/2019090427

**Published:** 2019-11-14

**Authors:** Nai-Wen Chang, Yi-Ping Huang

**Affiliations:** 1 Department of Biochemistry, College of Medicine, China Medical University Taichung 404 Taiwan; 2 Department of Physiology, College of Medicine, China Medical University Taichung 404 Taiwan

**Keywords:** Next-generation sequencing, Oral cancer, PPARα, RNA degradation pathway

## Abstract

Background: The activation of peroxisome proliferator-activated receptor alpha (PPARα) has been shown to reprogram tumor metabolism and exhibits great potential for treating anti-oral tumorigenesis.

Methods: In this study, we used a pathway-based strategy to explore possible functional pathways involved in the anticancer activity of PPARα in oral cancer cells through next-generation sequencing (NGS) and bioinformatic approaches.

Results: We found that 3919 genes were upregulated and 1060 genes were downregulated through PPARα activation. These genes were mainly involved in the proteasomal, mRNA surveillance, spliceosomal, RNA transport, and RNA degradation pathways, as indicated by GO and KEGG enrichment analysis. Importantly, a total of 13 upregulated genes in the RNA degradation pathway were identified including 3 core exosome factor genes (*RRP43, RRP42,* and *CSL4*), 2 TRAMP complex genes (*TRF4* and *Mtr4*), 2 exosome cofactor genes (*RRP6* and *MPP6*), 2 CCR4-NOT complex genes (*CNOT2* and *CNOT3*), 2 Ski complex genes (*SKI2* and *Ski3*), 1 decapping complex gene (*EDC4*), and 1 gene involved in 5’ exoribonuclease activity (*XRN1*).

Conclusion: Our findings suggest that the activation of PPARα to upregulate the RNA degradation pathway might provide a new strategy for oral cancer treatment.

## 1. Introduction

The diagnosis of oral cancer at an early stage provides an approximately 80% survival rate, while diagnosis at a late stage decreases the survival rate to 20% [1, 2]. The prognosis of patients is poor even after treatment with targeted and chemotherapeutic drugs [2]. Unfortunately, the molecular mechanisms underlying the recurrence of oral squamous cell carcinoma remain unclear.

Peroxisome proliferator-activated receptor alpha (PPARα) is known to regulate the expression of genes involved in lipid and glucose metabolism, inflammatory/vascular pathways, and tumor activity [3–7]. Our previous studies showed that the activation of PPARα through fenofibrate was involved in several anti-oral cancer activities in both animal and cell culture models including (a) the suppression of the development of the preneoplastic lesion into oral squamous cell carcinoma [8], (b) the downregulation of mTOR activity by TSC1/2-dependent signaling through the stimulation of AMPK and the repression of Akt [9, 10], (c) the regulation of the expression of genes related to mitochondrial energy metabolism [6], and (d) the inhibition of oral cancer cell proliferation and the induction of metabolic reprogramming by switching the energy production way from the Warburg effect to mitochondrial oxidative phosphorylation [11]. Thus, we suggested that the mechanisms by which PPARα activation suppressed tumor progression might modulate energy metabolism through the downregulation of the Warburg effect [11]. However, the functional evidence for the PPARα response described above is limited to tumor metabolism and energy production. In PPARα-mediated anti-oral tumorigenesis, it is possible that some oncogenic pathways or alterations have yet to be described.

Recently, several next-generation sequencing (NGS) studies of oral cancer have been conducted, and a number of gene mutations have been correlated with the risk of oral cancer [12, 13]. However, the method by which gene mutations modulate oral cancer behavior is mostly unknown. To explore the biological behavior of cancer, cancer cell lines are widely used as tools and provide a platform for further studies of cancer treatment and drug discovery. In this study, we used a pathway-based strategy to identify possible functional pathways involved in the anticancer activity of PPARα in oral cancer cells. SAS cells were treated with or without fenofibrate to activate PPARα and were collected for next-generation sequencing studies.

## 2. Methods

### 2.1. Cell culture

The SAS human oral cancer cell line used in this study was authenticated using the Promega GenePrint 10 System by Genelabs (Taipei, Taiwan) to confirm that no contamination was found [9]. The SAS cells were treated with 0.1% DMSO (control group) or 50 μM fenofibrate (PPARα group) (Sigma-Aldrich Corp., USA) for 24 hours as previously described [9]. Then, the cells were collected for total RNA extraction and expression profiling. All data were collected from three independent experiments.

### 2.2. RNA extraction and sequencing

Total RNA from each sample was extracted using RNeasy Mini Kit (Qiagen) as previously described [14]. The quality of the extracted RNA was confirmed by an Agilent 2100 Bioanalyzer (Agilent Technologies) and a NanoDrop (Thermo Fisher Scientific Inc.), respectively. Next-generation sequencing library were prepared according to the manufacturer’s protocol (NEBNext® Ultra™ RNA Library Prep Kit, Illumina®) [14].

### 2.3. Quality control and expression analysis

To obtain high-quality and clean data, technical sequences with a lower quality were removed as previously described [14]. Gene expression calculation was performed with Cuffdiff (v 2.2.1), which calculated FPKM (fragments per kilobases per million reads) [14]. The expression levels of all genes under different experimental conditions were compared by FPKM profiles.

### 2.4. Differential expression analysis

The DESeq Bioconductor package, which uses a model based on a negative binomial distribution, was used for differential expression analysis as previously described [15, 16]. The results from DESeq2 analysis were further analyzed to identify genes exhibiting significant differential expression according to the criteria of a fold change greater than 2 and a FDR (false discovery rate) less than 0.05. After adjustment by Benjamini and Hochberg’s approach to control for the FDR, the Wald test was used to analyze the differences. Differences were considered significant at *P* < 0.05 [14].

### 2.5. GO and KEGG enrichment analysis

We first used GO (Gene Ontology) analysis to map all the differentially expressed genes to the GO database and then calculated the number of differential genes for each term. The GO terms enriched in differential genes were determined using a hypergeometric test against the genomic background. In addition, the KEGG (Kyoto Encyclopedia of Genes and Genomes) is a collection of databases that involve chemical substances, drugs, genomes, biological pathways, and diseases [17]. Pathway enrichment analysis was based on KEGG pathway units and used a hypergeometric test to find which pathways were significantly enriched for differentially expressed genes against the transcriptome background. Differences were considered significant at *P* < 0.05.

## 3. Results

### 3.1. Determination of differential gene expression

**Fig. 1:**
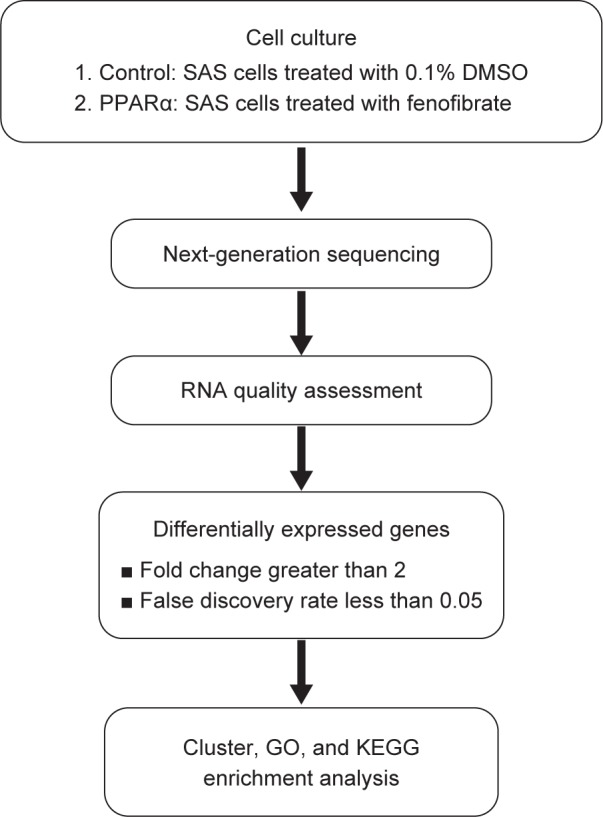
Flowchart of the study design. The SAS cells were treated with 0.1% DMSO (control) or 50 μM fenofibrate for 24 hours. Then, the cells were collected for total RNA extraction and expression profiling. The results from DESeq2 analysis were further analyzed to identify genes with significantly differential expression according to the criteria of a fold change greater than 2 and a false discovery rate less than 0.05. Differentially expressed genes were selected for further cluster, GO, and KEGG enrichment analysis.

**Fig. 2:**
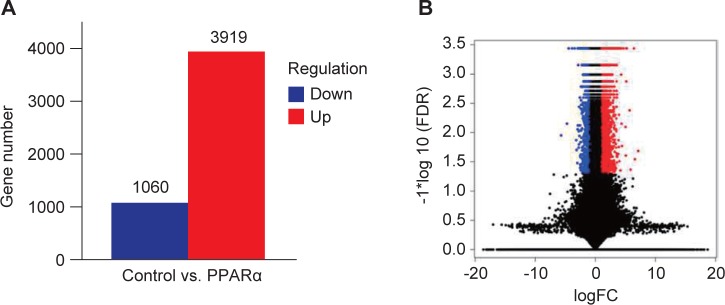
Summary of the numbers of differentially expressed genes that are significantly up- or downregulated compared to their expression in control cells. (A) Bar graph of genes significantly up- or down-regulated in PPARα-induced cells compared to their expression in control cells. (B) Differential expression volcano plot, in which red dots represent genes that are significantly upregulated and blue dots represent those that are significantly downregulated compared to their expression in control cells. The X-axis indicates the log2 FC of gene expression. The Y-axis indicates the statistical significance of differential expression as log10 (FDR). FC: fold change; FDR: false discovery rate.

The RNA expression profiles following PPARα activation in SAS cells were obtained from NGS. The workflow of the analysis is summarized in Fig. 1. The results from DESeq2 analysis were further analyzed to identify genes exhibiting significantly differential expression according to the criteria of a fold change greater than 2 and a FDR less than 0.05. Gene expression in the control group was used as a baseline to determine the up- or downregulation of gene expression in PPARα-induced cells. We found that 3919 genes were upregulated and 1060 genes were downregulated following PPARα activation (Fig. 2A). The numbers of differentially expressed genes are displayed as a volcano plot in Fig. 2B.

### 3.2. GO classification of differentially expressed genes

**Fig. 3:**
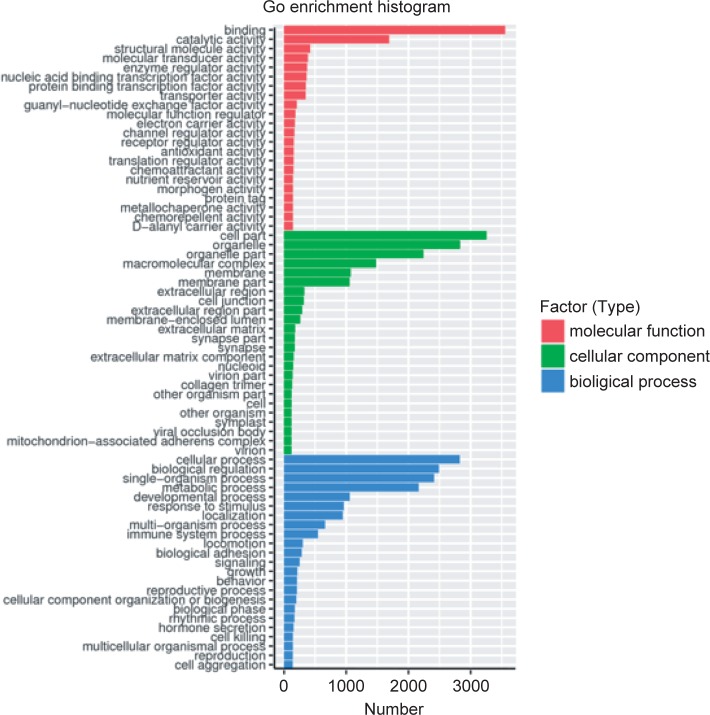
GO enrichment analysis of differentially expressed genes that are significantly different between the control and PPARα-induced cells. The X-axis indicates the number of differentially expressed genes in a GO category. The Y-axis indicates the GO categories. Color coding is used to distinguish the molecular function, cellular component and biological process categories.

Next, a total of 4979 differentially expressed mRNAs between the control and PPARα groups were determined by GO enrichment analysis. The results showed that these genes were involved in molecular functions, biological processes, and cellular components (Fig. 3). In the molecular function category, the most abundant subcategories were catalytic activity and cofactor binding. The top 5 most prominent enrichment subcategories involved in the biological process category were cellular processes, biological regulation, single-organism processes, metabolic processes, and developmental processes. In the cellular component category, the most abundant subcategories were cell parts, organelles, organelle parts, and macromolecular complexes.

### 3.3. Scatter plot of differential gene KEGG enrichment

**Fig. 4:**
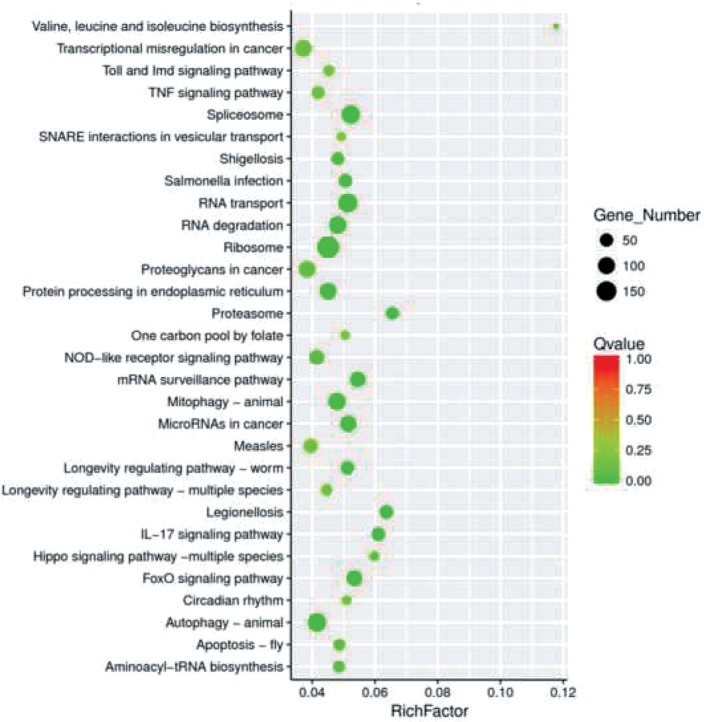
Scatter plot of differential gene KEGG enrichment. The X-axis indicates the Rich factor. The Y-axis specifies the KEGG pathways. The size of the dot is positively correlated with the number of differentially expressed genes in the pathway. Color coding indicates different q value ranges. The Rich factor indicates the ratio of the number of differentially expressed genes in the pathway to the total number of genes in the pathway. A greater Rich factor indicates a greater degree of enrichment. The Q value is the *P* value after multiple hypothesis testing and ranges between 0 and 1; the closer the Q value is to zero, the more significant the enrichment is.

To understand the mechanism of the differential expression of the mRNAs described above, we implemented KEGG pathway enrichment analysis. The degree of KEGG enrichment was evaluated by the q value, the rich factor, and the number of genes enriched in the pathway [17]. The top 30 pathways with the most significant enrichment after screening are listed in Fig. 4. Remarkably, these mRNAs were enriched in the proteasomal, mRNA surveillance, spliceosomal, RNA transport, and RNA degradation pathways.

### 3.4. Upregulated genes involved in the RNA degradation pathway through PPARα activation

It is known that mRNA degradation pathway plays a crucial role in cancer gene expression and cellular functions [18, 19]. The eukaryotic RNA exosome catalyzes RNA degradation and cooperates with cofactors such as TRAMP, CCR4-NOT, and Ski complexes to specifically target transcripts for 3’ to 5’ degradation, while 5’ to 3’ mRNA degradation is initiated by decapping complexes [20, 21]. We next identified genes in the RNA degradation pathway that exhibited significantly differential expression, which are shown in Table 1. According to the criteria of a fold change greater than 2 and a FDR less than 0.05, a total of 13 upregulated genes were identified in PPARα-induced cells. We found that 3 core exosome factor genes (*RRP43, RRP42,* and *CSL4*), 2 TRAMP complex genes (*TRF4* and *Mtr4*), 2 exosome cofactor genes (*RRP6* and *MPP6*), 2 CCR4-NOT complex genes (*CNOT2* and *CNOT3*), and 2 Ski complex genes (*SKI2*, and *Ski3*) were involved in 3’ to 5’ mRNA degradation. In addition, the *EDC4* and *XRN1* genes were involved in 5’ to 3’ mRNA degradation [26]. The loss of any one subunit of the nine genes listed above is lethal [26]. Our study showed the significant upregulation of the expression levels of *RRP43, RRP42,* and *CSL4* in PPARα-induced cells. Although the molecular mechanisms for this remain unclear, mutations in the RNA exosome gene *RRP43* (*EXOSC8*) have been shown to be associated with pontocerebellar hypoplasia type 1c [28].

**Table 1:** List of upregulated genes in the mRNA degradation pathway following PPARα activation.

Gene Id	Gene Name	Log2 Fold Change	*P*-value Adjusted^a^	KEGG BRIT(mRNA Degradation Factors)	Localization
**3’→5’ decay**					
ENSG00000120699	*RRP43, EXOSC8*	1.216	0.0003653	Core exosome factor	Nucleus/Cytoplasm
ENSG00000075914	*RRP42, EXOSC7*	1.028	0.0013357	Core exosome factor	Nucleus/Cytoplasm
ENSG00000171311	*CSL4, EXOSC1*	1.348	0.0003653	Core exosome factor	Nucleus/Cytoplasm
ENSG00000112941	*TRF4, PAPD7*	2.015	0.0003653	TRAMP complex	Nucleus
ENSG00000039123	*Mtr4, SKIV2L2*	1.727	0.0325274	TRAMP complex	Nucleus
ENSG00000171824	*RRP6, EXOSC10*	2.019	0.0003653	Exosome cofactor	Nucleus/Cytoplasm
ENSG00000135698	*MPP6, MPHOSPH6*	1.119	0.0075373	Exosome cofactor	Nucleus
ENSG00000111596	*CNOT2, NOT2*	2.235	0.0003653	Ccr4-NOT complex	Cytoplasm
ENSG00000088038	*CNOT3, NOT3*	1.839	0.0003653	Ccr4-NOT complex	Cytoplasm
ENSG00000204351	*SKI2, SKIV2L*	1.773	0.0019390	Ski complex	Cytoplasm
ENSG00000198677	*Ski3, TTC37*	1.385	0.0003653	Ski complex	Cytoplasm
**5’→3’ decay**					
ENSG00000038358	*EDC4*	1.443	0.0062746	Decapping complex	Cytoplasm
ENSG00000114127	*XRN1, SEP1*	2.397	0.0298322	5’ Exoribonuclease	Cytoplasm

^a^ *P*-value is adjusted for multiple testing using Benjamini and Hochberg’s approach to estimate the false discovery rate.

## 4. Discussion

Our previous studies suggested an important role for PPARα in anti-oral cancer activity in both animal and cell culture models [8, 9]. In this study, a next-generation sequencing approach was used to determine differentially expressed genes in PPARα-induced SAS cells. The results showed that 3919 genes were upregulated and 1060 genes were downregulated through PPARα induction, and these genes were mainly involved in the proteasomal, mRNA surveillance, spliceosomal, RNA transport, and RNA degradation pathways, as shown by GO and KEGG enrichment analysis. Among the 5 candidate pathways, the relationship between the RNA degradation pathway and oral tumor progression remains unclear. Therefore, we selected the RNA degradation pathway for further analysis.

It is known that exosomes mediate communication within the tumor microenvironment and play important roles in tumor development, invasion, and metastasis [22, 23]. Therefore, targeting RNA degradation pathways by blocking the RNA exosome and its cofactors could be a critical strategy in oral cancer therapy. In this study, we confirmed that the mRNA degradation pathway might be involved in PPARα-induced anticancer activity. A total of 13 upregulated genes were identified in the RNA degradation pathway including 3 core exosome factor genes (*RRP43, RRP42,* and *CSL4*), 2 TRAMP complex genes (*TRF4* and *Mtr4*), 2 exosome cofactor genes (*RRP6* and *MPP6*), 2 CCR4-NOT complex genes (*CNOT2* and *CNOT3*), 2 Ski complex genes (*SKI2* and *Ski3*), 1 decapping complex gene (*EDC4*), and 1 gene involved in 5’ exoribonuclease activity (*XRN1*).

The eukaryotic RNA exosome can degrade or process RNA substrates in the 3’ to 5’ direction [24, 25]. The core of the RNA exosome complex is composed of three S1/KH RNA-binding proteins (CSL4, RRP4, and RRP40) and six RNase PH-like proteins (MTR3, RRP41, RRP42, RRP43, RRP45, and RRP46) [26, 27].

The eukaryotic RNA exosome functions in both the cytoplasm and the nucleus [21, 28]. Nuclear RNA exosomes can cooperate with cofactors such as the TRAMP complex (Trf4/5-Air1/2-Mtr4 polyadenylation), MPP6, RRP6, and the NNS complex (Nrd1-Nab3-Sen1) to perform RNA degradation [21, 28]. Our study showed that PPARα significantly upregulated the expression levels of the *TRF4, Mtr4, RRP6,* and *MPP6* genes, all four of which are known to be related to the RNA degradation pathway. Such differences provided a link between the exosomal contents and tumor progression. Recently, the MPP6 protein was reported to regulate the protumorigenic activity of SAA3, a member of the serum amyloid A apolipoprotein family, in pancreatic ductal adenocarcinoma [29].

The cytoplasmic mRNA degradation pathway begins with removing the poly(A) tail by the CCR4-NOT complex [30], followed by the degradation of the mRNA in the 3’ to 5’ or 5’ to 3’ directions. The nine core subunits of the CCR4-NOT complex are CNOT1, CNOT2, CNOT3, CNOT6, CNOT6L, CNOT7, CNOT8, CNOT9, and CNOT10 [31, 32]. Its biological functions are involved in regulation of mRNA stability, transcription, and translation [33, 34]. Our study showed that the *CNOT2* and *CNOT3* genes were upregulated by PPARα activation and that their expression might promote the anticancer activity of PPARα. Although how CNOT2 and CNOT3 function in the catalytic subunit during the oral cancer process remains unclear, a mutation in *CNOT3* is associated with non-small cell lung cancer and T-cell acute lymphoblastic leukemia [35, 36]. Studies from a breast cancer mouse model and human patient data demonstrate a negative correlation between *CNOT2* expression and tumor development and the CCR4-NOT complex was implicated in tumor cell metastasis [37, 38]. In addition, cytoplasmic mRNA degradation in the 3’ to 5’ direction is mainly catalyzed by exosomes and their cofactors such as the Ski complex (Ski2–Ski3–Ski8) and Ski7 [21, 28, 39]. In this study, we observed the upregulation of the *SKI2* and *Ski3* genes following PPARα activation. Mutations in the *SKI2* and *Ski3* genes have been shown to be associated with syndromic diarrhea/trichohepatoenteric syndrome [40, 41].

Finally, we confirmed that the expression levels of the *EDC4* and *XRN1* genes, which are involved in 5’ to 3’ RNA decay, were upregulated by PPARα. The mRNA degradation in the 5’ to 3’ direction is initiated by the decapping protein EDC4, and the mRNA is subsequently degraded by the exoribonuclease XRN1 [20, 42]. Zangari *et al*. showed that, in mammals, the XRN1 exoribonuclease specifically targets extracellular miR-223-3p and promotes transient epithelial-mesenchymal transition in human tumor cells [43].

Very little information is currently available concerning the degradation of mRNAs and oral cancer progression. The role of PPARα in the direct regulation of the mRNA degradation pathway is still unclear. Further studies are needed to explore the specificity of the effect of PPARα activation on the expression of genes involved in the RNA degradation pathway with PPARα knockdown or pretreatment with PPARα antagonists. Nevertheless, the identification of PPARα-targeted genes in the RNA degradation pathway may help to highlight treatment strategies for oral cancer.

In conclusion, next-generation sequencing analysis revealed that a total of 13 genes (*RRP43, RRP42, CSL4, TRF4, Mtr4, RRP6, MPP6, CNOT2, CNOT3, SKI2, Ski3, EDC4,* and *XRN1*) involved in the mRNA degradation pathway were upregulated by PPARα activation in oral cancer cells. Therefore, we suggest that the activation of PPARα to enhance the mRNA degradation pathway might provide a new strategy for oral cancer therapy.
